# Levothyroxine Intake Timing During Ramadan in Patients With Hypothyroidism: A Systematic Review and Meta-Analysis of Research Findings

**DOI:** 10.7759/cureus.108163

**Published:** 2026-05-03

**Authors:** Ahlam Alzenaidi, Faisal Alzenaidi, Abdulaziz Aloraini, Khalid M Alsulaim, Abdulsalam Aloraini, Turki Alharbi, Ahmed Alajlan, Lamis Alwabli

**Affiliations:** 1 Department of Medicine, College of Medicine, Qassim University, Buraydah, SAU; 2 Department of Medicine, College of Medicine, Qassim University, Unayzah, SAU

**Keywords:** fasting, hypothyroidism, levothyroxine, ramadan, systematic review and meta-analysis

## Abstract

Daily medication is challenging in the Islamic holy month of Ramadan, particularly for patients on levothyroxine replacement therapy for hypothyroidism. Ramadan necessitates significant alterations to daily dietary habits and medication schedules, which may impact drug absorption and efficacy. This study aims to synthesize findings from existing research on the optimal timing of levothyroxine intake during Ramadan and to compare these results with established recommendations for nonfasting periods. By searching MEDLINE, PubMed, Google Scholar, Web of Science, and CENTRAL Cochrane Library databases from inception to May 2025, a systematic review and meta-analysis of randomized controlled trials (RCTs) and observational studies were conducted to evaluate the optimal timing of levothyroxine intake during Ramadan in patients with hypothyroidism. The study protocol was registered on PROSPERO (CRD420261284016). The primary outcome was thyroid-stimulating hormone (TSH) levels after Ramadan fasting. We used the standardized mean difference (SMD) with corresponding 95% confidence intervals (CIs) to synthesize results in the absence of heterogeneity. The risk of bias was assessed using Cochrane Risk of Bias 2 and Quality Assessment of Diagnostic Accuracy Studies 2 tools for RCTs and observational studies, respectively. We screened 214 records and included nine studies. A small but statistically significant increase was noted in post-Ramadan TSH levels compared with the prefasting levels (SMD = 0.29; 95% CI = 0.15-0.42; Z = 4.17; p < 0.001). However, most patients (79.8%) can maintain euthyroidism with different intake times (85.3%) if adherence is optimal. The results highlight various outcomes across different timings and the importance of patient adherence. Ultimately, rather than the timing of levothyroxine intake, adherence is the most likely factor determining the adequacy of thyroid function during and after Ramadan fasting.

## Introduction and background

Hypothyroidism, a prevalent endocrine disorder affecting a significant global population, necessitates precise and consistent levothyroxine administration to achieve and maintain biochemical euthyroidism [1]. However, the efficacy of oral levothyroxine is highly dependent on factors such as absorption, metabolism, and patient adherence, and dose adjustments are frequently necessary in clinical practice, which is shown to consume more healthcare resources [2]. This is particularly challenging for patients observing Ramadan, a period of fasting from dawn until dusk, which significantly alters dietary intake and medication schedules [3]. Consequently, the timing of levothyroxine administration during Ramadan requires careful consideration to ensure optimal absorption and sustained therapeutic efficacy, especially given that various factors, including gastrointestinal conditions and concomitant medications, can impair its bioavailability [4]. In addition, daily routines significantly change during Ramadan, impacting not only dietary habits but also the timing of medication intake. This shift necessitates a thorough examination of how the altered fasting and feeding cycles inherent to Ramadan influence the absorption and bioavailability of levothyroxine, a drug known for its narrow therapeutic index and sensitivity to gastrointestinal conditions and concomitant substances [3]. The complexities of managing levothyroxine during this period are further compounded by scenarios of noncompliance or malabsorption, which can lead to refractory hypothyroidism, characterized by persistently elevated thyrotropin levels despite seemingly adequate dosing [5]. The inherent challenges in maintaining stable thyrotropin levels during Ramadan necessitate a thorough understanding of the absorption dynamics of levothyroxine, particularly in the context of altered fasting and feeding cycles [6]. Ultimately, understanding the multifactorial influences on levothyroxine pharmacokinetics during Ramadan is crucial for clinicians to provide personalized care that balances religious observances with optimal therapeutic management, thereby preventing adverse clinical outcomes. Several studies have investigated the optimal timing for levothyroxine administration in hypothyroid patients during the month of Ramadan, with the goal of maintaining stable thyroid hormone levels while fasting.

This review focuses specifically on identifying studies that compare the efficacy of taking levothyroxine at Suhoor (predawn meal) vs. Iftar (postdusk meal) or bedtime and evaluating the impact of these timings on thyroid hormone profiles and patient outcomes. This analysis will also delve into the methodological approaches employed by these studies, assessing their robustness and the generalizability of their findings while identifying gaps in the current literature to guide future research directions. Finally, this review addresses the critical role of physician-patient communication in preempting and managing potential complications arising from modified levothyroxine regimens during this culturally significant period, thereby fostering better health outcomes.

## Review

Methods

This study was conducted in accordance with the current Preferred Reporting Items for Systematic Reviews and Meta-Analysis 2020 guidelines [7]. The process followed the Cochrane Collaboration statement. We registered the study protocol on PROSPERO (CRD420261284016).

Systematic Search Strategy

This systematic review was conducted in PubMed, Web of Science, MEDLINE, Google Scholar, and CENTRAL Cochrane Library databases for publications and ongoing trials.

PICO framework (population, intervention, comparator, and outcome) was used as follows: P - hypothyroid patient during Ramadan; I - levothyroxine; C -different levothyroxine regimen; O: adherence, thyroid-stimulating hormone (TSH) level, and clinical outcome during Ramadan.

The search was conducted from March to May 2025. The databases were searched utilizing the keywords “hypothyroidism”, “OR”, “hypothyroid”, “AND”, “levothyroxine”, “OR, “thyroid hormone”, and “AND, “Ramadan”. We identified all the studies’ titles, abstracts, and methods using Rayyan software (Rayyan Systems Inc., Cambridge, MA) and checked them for eligibility.

All citations and references in the included articles were identified and checked for eligibility for inclusion.

Screening and Eligibility Criteria

Two authors conducted the title and abstract screening according to the predetermined criteria. After the initial screening, the full texts of the included studies were screened by two authors. During both stages, two authors were involved in solving any conflicts. All randomized controlled trials (RCTs) and observational studies focusing on adherence to levothyroxine among hypothyroid patients during Ramadan, including those that provide data on pre- and posttreatment TSH levels, clinical outcomes, and different levothyroxine dosages and timings, were included. We excluded studies involving pregnant and lactating patients, patients with multiple medications that interfere with levothyroxine absorption, and patients younger than 18 years.

Data Extraction

Data were extracted using the Rayyan software. The extracted data included the study characteristics, baseline information of the included patients, intervention details, outcomes of interest (TSH level before and after Ramadan fasting), and timing of levothyroxine intake during the month of Ramadan. After the initial extraction, another author revised the data for any discrepancies.

Quality Assessment of the Studies' Risk of Bias

Three authors independently assessed risk of bias using the Cochrane Revised Risk of Bias Tool for Randomized Trials (Risk of Bias 2, RoB2) and the Quality Assessment of Diagnostic Accuracy Studies 2 (QUADAS-2) for observational studies [8,9]. In situations where conflict arose, a fourth reviewer was consulted to resolve the disparity. In the RoB2 tool, the domains judged include selection bias, performance bias, attrition bias, and detection bias. The overall risk of bias options for each category were low risk, unclear risk, and high risk of bias. With respect to the QUADAS-2 tool, four key domains were assessed: patient selection, index test, reference standard, flow, and timing. Each domain was evaluated for risk of bias, and the first three domains were also assessed for concerns regarding applicability.

Statistical Analysis

All the quantitative analyses and meta-analytical computations were conducted using Review Manager (RevMan) version 5.4.0 (The Cochrane Collaboration, London, UK). The pooled effect size was expressed as the standardized mean difference (SMD) with corresponding 95% confidence intervals (CIs). Statistical heterogeneity was assessed using the Cochran Q test and quantified with the I² statistic, with values <25% indicating low heterogeneity. A fixed-effects model was applied because of the low observed heterogeneity. Statistical significance was determined using the Z test, with a two-tailed P value <0.05 considered statistically significant. The risk-of-bias assessment for RCTs was conducted using the Cochrane Risk of Bias tool (Rob 2), evaluating domains including random sequence generation, allocation concealment, blinding, incomplete outcome data, and selective reporting. For nonrandomized observational studies, methodological quality was assessed using the QUADAS-2 tool.

Results

Study Selection

**Figure 1:**
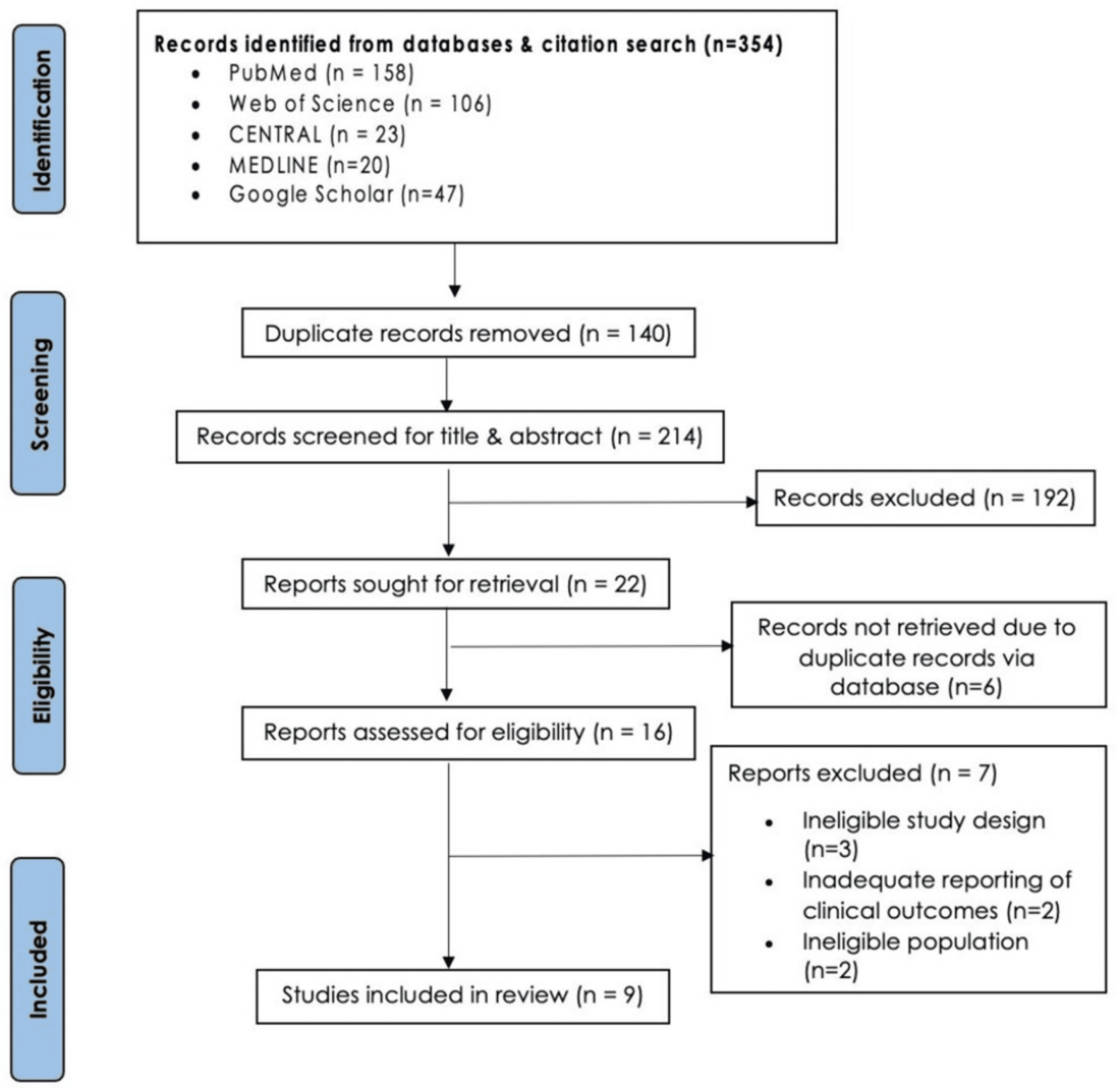
PRISMA 2020 flow diagram illustrating study identification, screening, eligibility, and inclusion for the review of levothyroxine timing during Ramadan PRISMA: Preferred Reporting Items for Systematic Review and Meta-Analysis Source: [7]

A total of 354 studies were identified after the previously mentioned databases were searched, of which 214 studies remained after duplicate records were removed (140 studies). After the titles and abstracts were screened, 22 studies were retrieved. Following full-text screening, only nine studies that met the inclusion criteria were included in the data extraction process [10-18]. The flowchart of the study selection process is presented in Figure 1.

Study Characteristics

The final sample comprised nine studies, including three RCTs and six observational studies. The geographic distributions of the study population were as follows: Egypt (two), Turkey (one), and Asia (Saudi Arabia (two), Pakistan (one), Malaysia (one), Iran (one), and India (one)). All included studies were published in English. Across studies, the population consisted of adults with hypothyroidism who were observing Ramadan, with variable levothyroxine intake timing (predawn Suhoor, postdusk Iftar, and/or bedtime). Reported outcomes include thyroid function (primarily TSH and free T4), adherence measures, and, less consistently, safety/adverse events. The main characteristics of the nine included studies are reported in Table 1.

**Table 1:** The main characteristics of the nine included studies FT4: free T4; TSH: thyroid-stimulating hormone; DTC: differentiated thyroid cancer Source: [10-18]

Study	Study type	Region/country	Measured outcomes	Sample size (n)	Outcome (results)	Conclusion
Alzahrani et al. [10]	Randomized controlled trial	Saudi Arabia	FT4, TSH (before and after Ramadan)	69	The median thyrotropin (TSH) level and the number of patients with euthyroidism, subclinical hypothyroidism (SHypo), or subclinical hyperthyroidism (SHyper) at the start of Ramadan (baseline) were similar between the two groups (P = 0.69 and P = 0.65, respectively). Arm A had 17 (51.5%), 3 (9.1%), and 13 (39.4%) patients with euthyroidism, SHyper, and SHypo at the conclusion of Ramadan, while arm B had 17 (47.2%), 14 (38.9%), and 5 (13.9%) patients, respectively (P = 0.005). At the conclusion of Ramadan, the mean ± SD TSH levels in arms A and B were 5.6 ± 6.0 and 1.67 ± 2.6 mU/L, respectively (P = 0.0001)	There were higher instances of SHypo in arm A and SHyper in arm B, but no overt thyroid malfunction appeared. In 86% of cases, Arm B produced acceptable TSH levels (normal or slightly suppressed), which may be a better strategy, particularly for patients who require TSH suppression (e.g., DTC)
Elsherbiny [11]	Pilot prospective study	Egypt	FT4, TSH (before and after Ramadan)	124	Regarding adherence, post-Ramadan TSH, and post-Ramadan thyroid status, there were no appreciable variations between the two groups. Adherence rates were 90.9% in group 1 and 88.5% in group 2 (P = 1.000). TSH levels after Ramadan were 1.9 ± 1.5 mIU/L in group 1 and 2 ± 1.6 mIU/L in group 2 (P = 0.809). After Ramadan, 81.8% of group 1 and 82.3% of group 2 were euthyroid (P = 0.209)	For hypothyroid individuals who want to fast throughout Ramadan, administering L-T4 twice or three times a week produced adherence and metabolic control comparable to conventional daily L-T4 in this pilot trial
Elsherbiny [12]	Prospective study	Egypt	FT4, TSH (before and after Ramadan)	292	The majority of patients, 249, or 85.3%, were adherent, while 43, or 14.7%, were nonadherent. TSH was 1.60 ± 0.96 mIU/L prior to Ramadan and 2.13 ± 1.88 mIU/L following Ramadan (P = 0.001). After Ramadan, 233 patients (79.8%) remained euthyroid, while 59 individuals (20.2%) had dysthyroidism. TSH levels before and after Ramadan were substantially associated (P < 0.001). Post-Ramadan TSH was 3.57 ± 3.11 mIU/L in nonadherent individuals and 1.88 ± 1.44 mIU/L in adherent patients (P < 0.001)	Although post-Ramadan TSH significantly increased in well-controlled hypothyroid individuals who fasted during Ramadan, 80% of these patients remained euthyroid. Pre-Ramadan TSH and adherence are associated with post-Ramadan TSH and euthyroidism
Ghaffar et al. [13]	Cohort study	Pakistan	FT4, TSH (before and after Ramadan)	44	Prior to Ramadan, the average TSH was 2.85 ± 1.34 m IU/L. After Ramadan, the TSH rose to 3.84 ± 2.46 m IU/L, with a significant difference (P = 0.014). Thirty-six patients had TSH levels in the normal range prior to Ramadan, 8 had elevated TSH levels, and 20 had elevated TSH levels after Ramadan. When thyroxine was taken less than half an hour before eating, there was an increase in TSH levels. TSH levels and serum cholesterol levels were positively correlated	Participants taking thyroxine less than half an hour before or after sehari have higher TSH levels, and there is a positive connection between TSH levels and lipid markers
Karoli et al. [14]	Prospective observational study	India	FT4, TSH (before and after Ramadan)	47	TSH varied generally between 0.6 and 8 (2.4 ± 2.1) mIU/L. We discovered that 18/47 individuals had a 2 mIU/L variation in TSH compared to their pre-Ramadan assessment, while 29/47 patients had higher TSH readings (≥2 mIU/L). Concomitant diseases and the meal-levothyroxine interval differed significantly between the two groups. The meal-levothyroxine interval and TSH fluctuation were significantly correlated (r = -0.32, P = 0.01)	Patients with hypothyroidism may be able to take their medication before bedtime during Ramadan or at other times, but there should be at least two hours between meals. Pregnant women, the elderly, and people with osteoporosis are among those who require frequent and careful monitoring in order to maintain TSH in a limited range
Or Koca et al. [15]	Prospective observational study	Turkey	FT4, TSH (before and after Ramadan)	97	Patients' median blood levels of TSH were 2.19 mIU/L before fasting and 2.73 mIU/L afterward. After Ramadan, serum TSH levels were substantially higher than before (P = 0.004)	In individuals with hypothyroidism who are fasting, a significant increase in serum TSH levels during Ramadan is shown, but no significant change in serum-free thyroxine (fT4) levels. For some hypothyroid individuals who want to fast, it can be appropriate to take precautions by slightly increasing the LT4 dose prior to Ramadan
Mahzari et al. [16]	Randomized controlled trial	Saudi Arabia	FT4, TSH (before and after Ramadan)	87	Patients taking levothyroxine three to four hours after iftar had lower compliance. Furthermore, most patients who did not have a specific indication took levothyroxine half an hour prior to iftar. Following Ramadan, there was a statistically significant rise in TSH (P = 0.006) and FT4 (P = 0.044) levels. Levothyroxine dosage and the cause of hypothyroidism (Hashimoto's; postthyroidectomy; as opposed to postradioactive iodine) significantly affected FT4 levels in multivariate analysis. On the other hand, there was no significant correlation found between any variable and TSH levels. TSH and FT4 levels were not significantly impacted by the timing of levothyroxine consumption during Ramadan	Following Ramadan, TSH and FT4 were significantly elevated. However, neither TSH nor free T4 levels were affected by the timing of levothyroxine ingestion. Therefore, depending on their preferences, hypothyroid patients may take levothyroxine 30 minutes or 3-4 hours after iftar without eating for an hour
Zaboon et al. [17]	Prospective study design	Iraq	FT4, TSH (before and after Ramadan)	50	TSH levels before and after Ramadan had P values of 0.18, 0.75, and 1.0 for pre-suhoor, pre-iftar, and post-iftar, respectively. Patients with regulated TSH before Ramadan maintained control (8/10 pre-suhoor, 8/12 pre-iftar, 4/6 post-iftar), whereas patients with uncontrolled TSH before Ramadan improved (7/10 pre-suhoor, 6/8 pre-iftar, 2/4 post-iftar). The groups did not, however, vary significantly (P = 0.75 and 0.67)	Patients receiving L-thyroxine at pre-iftar, post-iftar, or pre-suhoor times during Ramadan showed no discernible variations in TSH management
Zakaria and Shahar [18]	Pilot randomized controlled trial	Malaysia	FT4, TSH (before and after Ramadan)	18	The majority (66.7%) had hypothyroidism as a result of radioiodine treatment. Thyroid hormone levels for the weekly arm did not significantly change at the conclusion of the research. The daily arm did, however, show a significant increase in TSH (TSH w0 1.8 (0.23, 5.57) vs. w4 3.65 (0.45, 16.1); P = 0.011). Despite a considerable increase in fT4 within 24 hours of weekly dosage (fT4 w0 13.21 (8.19, 14.63) vs. w2 17.43 (12.38, 22.55); P = 0.011), no hyperthyroid or cardiac toxicities were noted. Each subject had no adverse effects and was euthyroid. During Ramadan, the majority of patients (83.3%) favored a weekly dose	During Ramadan, a weekly levothyroxine dose seemed to be the most popular, safe, and effective dosing strategy

Synthesis of Results

**Figure 2:**
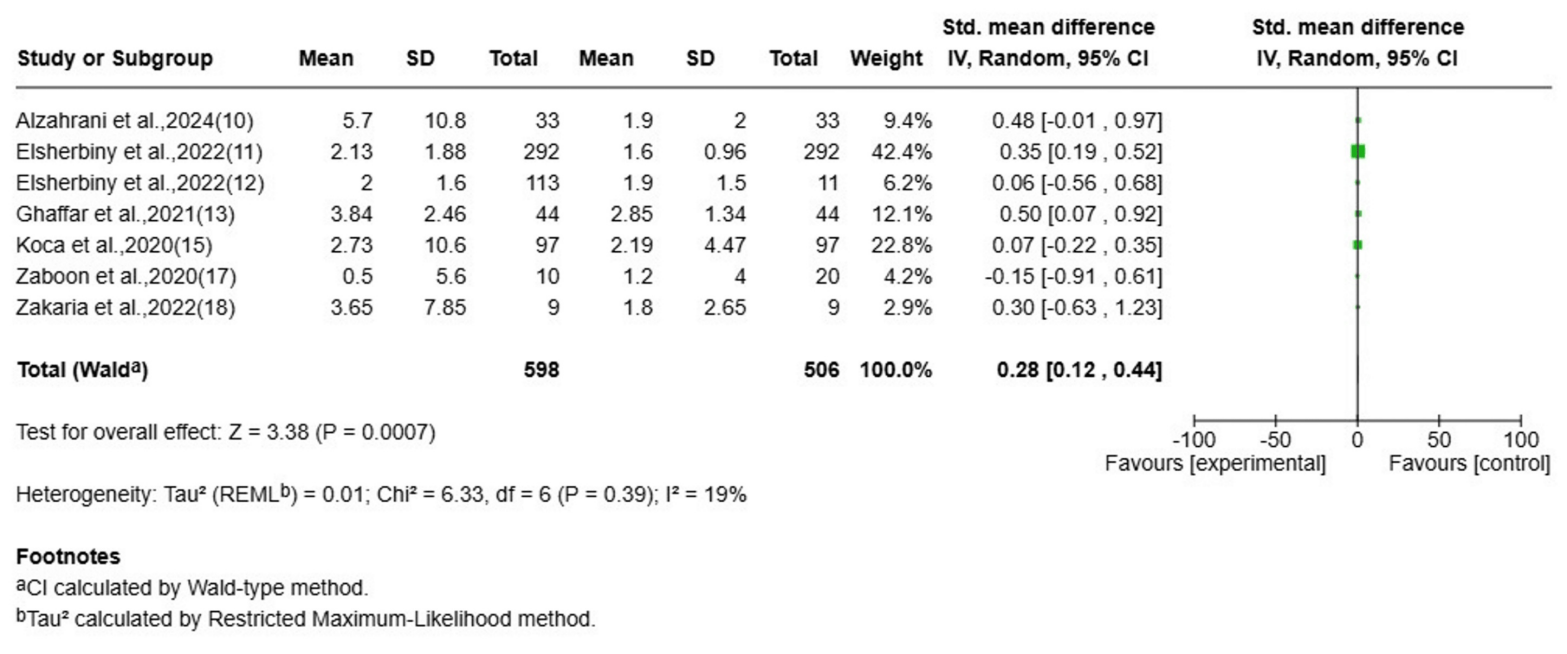
Pre- and post-Ramadan TSH (mIU/L) CI: confidence interval; REML: restricted maximum likelihood; TSH: thyroid-stimulating hormone Source: [10-13,15,17,18]

A meta-analysis was conducted of seven studies (n = 598) examining TSH levels before and after Ramadan fasting. There was a small but statistically significant increase in TSH after Ramadan (SMD = 0.29; 95% CI = 0.15-0.42; Z = 4.17; P < 0.001). Heterogeneity was low (I^2^ = 5%; P = 0.39), indicating consistency in the direction of effect across studies. These results indicated a small increase in TSH after Ramadan but did not establish causality or identify the underlying mechanism. In particular, they do not by themselves demonstrate changes in levothyroxine adherence or dosing efficacy (Figure 2).

The results of the comparative analysis revealed that a preemptive levothyroxine dose increase of 25-50 µg was more successful than just moving the time of levothyroxine consumption to the evening (5.6 ± 6.0 mIU/L) in reducing post-Ramadan TSH levels (1.67 ± 2.6 mIU/L). Nevertheless, this has increased the risk of subclinical hyperthyroidism to 38.9%. However, individuals who preferred a two- or three-weekly dose schedule (83.3%) exhibited noninferior TSH control (1.9 ± 1.5 mIU/L vs. 2.0 ± 1.6 mIU/L) for the daily dose regimen. The results also revealed that the majority of patients (79.8%) could maintain euthyroidism with different intake times (85.3%) if adherence was good (Table 2).

**Table 2:** Comparative analysis of the reported results of the included studies TSH: thyroid-stimulating hormone; RCT: randomized controlled trial Source: [10,12,18]

Study	Design	Time schedules and groups	TSH before Ramadan (mean ± SD)	TSH after Ramadan (mean ± SD)	Important occurrences/results (n/N or %)
Alzahrani et al. [10]	RCT	Arm A (evening): pre-Ramadan medication during dinner	~1.9 ± 2.0 (est.)	5.6 ± 6.0	17/33 (51.5%) euthyroid subclinical hypo: 39.4%, or 13/33
		Arm B (increased dosage): +25/50 µg of the pre-Ramadan dosage	~1.9 ± 2.0 (est.)	1.67 ± 2.6	17/36 (47.2%) are euthyroid. Subclinical hyper: 14 of 36 (38.9%)
Zakaria and Shahar [18]	Pilot RCT	Weekly dosage: 7 times a week before Sahur	1.8 (0.23, 5.57)	1.9 ± 1.5	15 of 18 patients (83.3%) prefer weekly dosing
		Daily dosage: take the drug at least two hours after eating	1.8 (0.23, 5.57)	2.0 ± 1.6	Weekly dosing preferred by patients: 15/18 (83.3%)
Elsherbiny [12]	Prospective	First routine: 60 minutes prior to Iftar	1.60 ± 0.96 (overall)	2.13 ± 1.88 (overall)	Patients who adhere: 249/292 (85.3%) post-Ramadan Euthyroid: 233/292 (79.8%)
		Regimen 2: 60 minutes before Suhur or 3-4 hours after Iftar	Not reported per group	Not reported per group	-
Other included studies	Pre-post	Different times (pooled)	1.6-2.85 (various SDs)	2.13-3.84 (various SDs)	Overall TSH increase: in the majority of studies, significant (P < 0.05)

Risk-of-Bias Assessment

Risk-of-Bias Assessment of the Included Nonrandomized Studies

Most of the included studies showed a moderate overall risk of bias on the basis of the QUADAS-2 evaluation. The "Patient Selection" and "Flow and Timing" domains showed a high risk of bias, whereas the "Index Test" and "Reference Standard" domains were primarily assessed as low risk. This pattern was consistent across all the studies. In addition to the high-risk trend observed in other investigations, one study by Karoli et al. was deemed to have a high overall risk of bias, primarily due to an ambiguous risk in the “Index Test” domain [14]. However, the findings may need to be presented cautiously because the research was generally of moderate methodological quality (Table 3).

**Table 3:** The risk of bias assessment using the methodological index for nonrandomized studies (QUADAS-2) assessment tool QUADAS-2: Quality Assessment of Diagnostic Accuracy Studies-2 Source: [11-15,17]

Study	Study design	Patients’ selection	Index test	Reference standard	Flow and timing	Overall risk of bias assessment per study
Elsherbiny et al. [11]	Pilot prospective study	High	Low	Low	High	Moderate risk
Elsherbiny et al. [12]	Prospective study	High	Low	Low	High	Moderate risk
Ghaffar et al. [13]	Cohort study	High	Low	Low	High	Moderate risk
Karoli et al. [14]	Prospective observational study	High	Unclear	Low	High	High risk
Or Koca et al. [15]	Prospective observational study	High	Low	Low	High	Moderate risk
Zaboon et al. [17]	Prospective study design	High	Low	Low	High	Moderate risk

Cochrane Risk-of-Bias Assessment for RCTs

**Figure 3:**
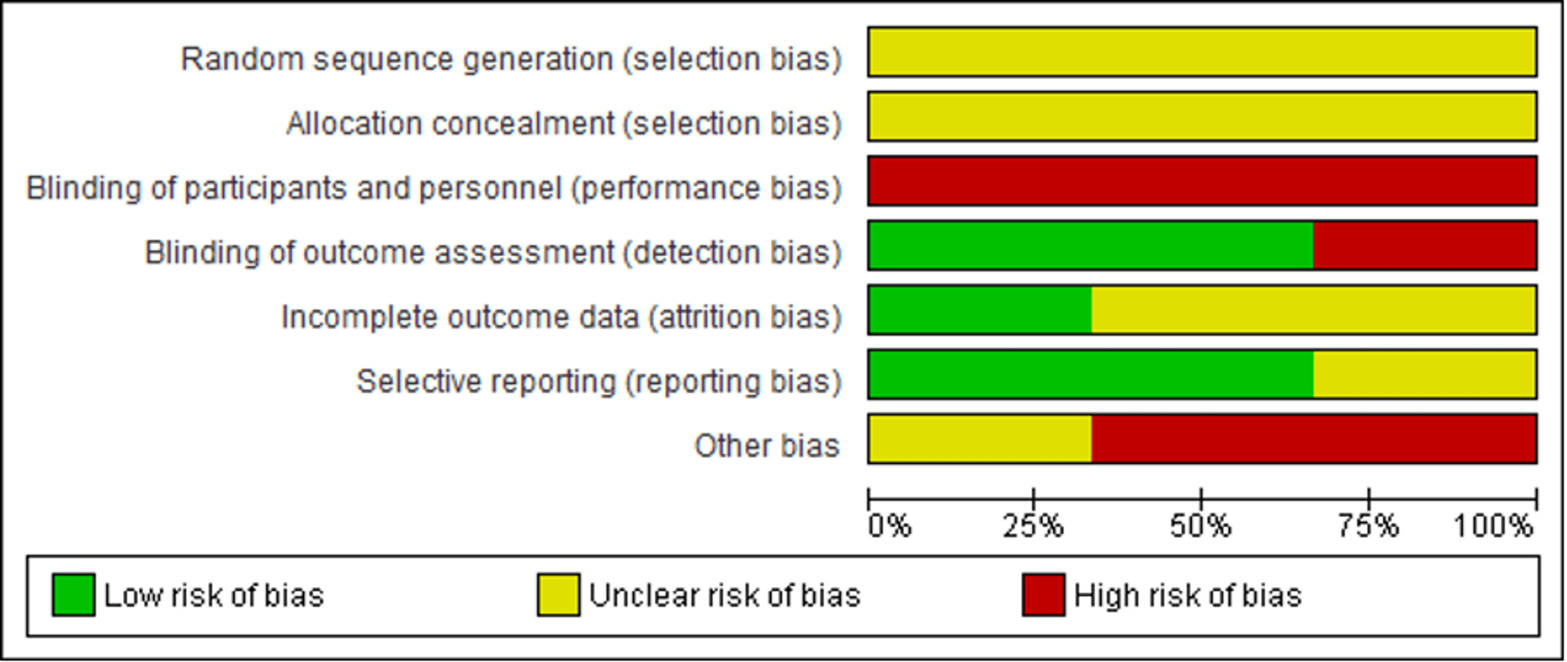
Risk-of-bias assessment domains according to Cochrane risk-of-bias for all randomized control studies (RoB2) tool RoB2: Risk of Bias 2 Source: [8]

**Figure 4:**
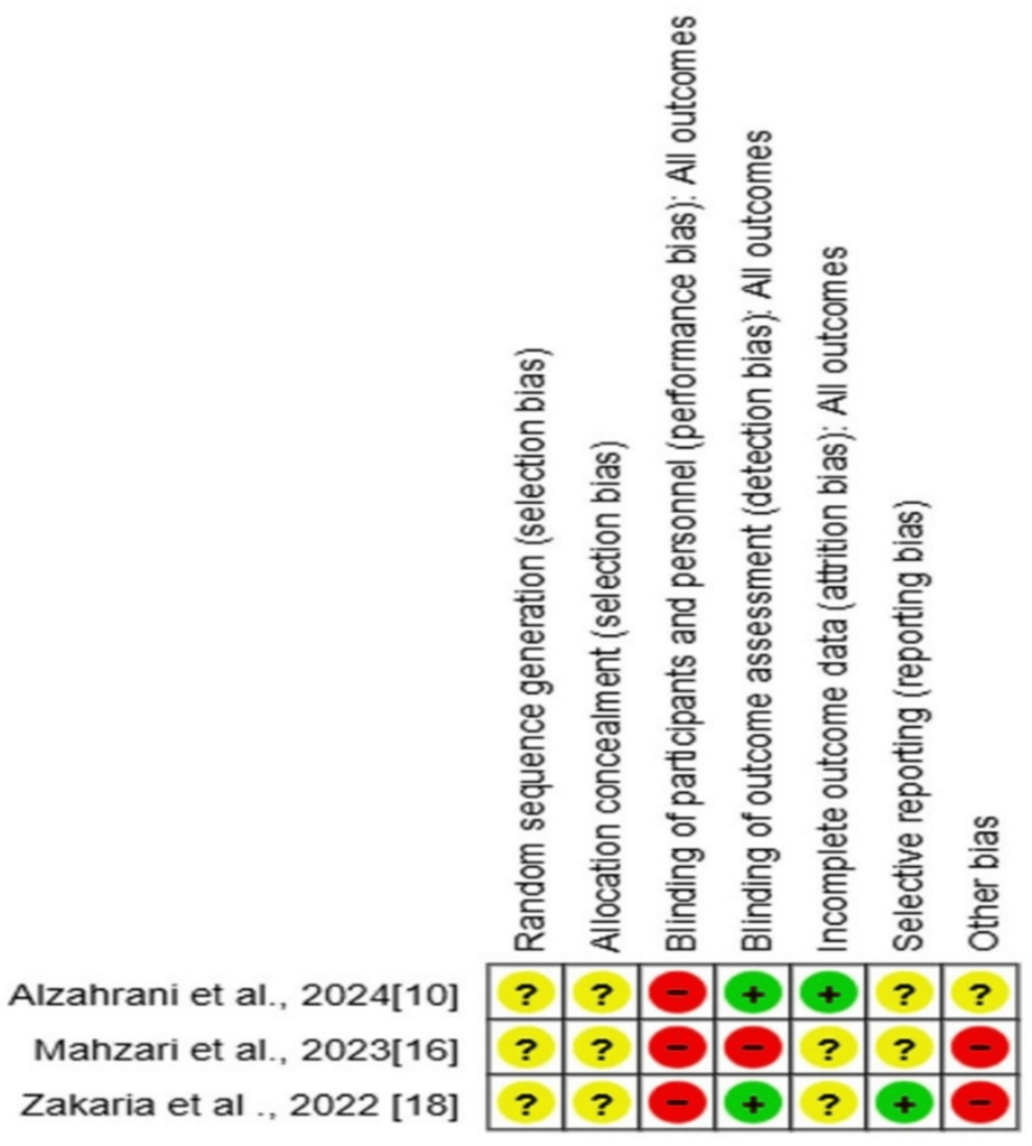
Risk-of-bias assessment of the studies investigating post-Ramadan TSH levels according to Cochrane risk-of-bias for randomized control studies TSH: thyroid-stimulating hormone Source: [10,16,18]

All evaluated RCTs had a high overall risk of bias. The most common risk was performance bias, which resulted from a lack of participant blinding. Allocation concealment was frequently unclear, although selection bias from random sequence creation was typically well controlled. The majority of trials had little detection bias, suggesting that outcome assessors were usually blinded. However, the attrition bias, reporting bias, and other possible biases of the research pool are still mostly unknown. In particular, of the three studies mentioned, Alzahrani et al. and Mahzari et al. revealed a low risk of bias for the majority of domains, whereas Zakaria and Shahar reported a significant risk of performance bias and an unclear risk for a number of other domains (Figures 3, 4) [10,16,18].

Discussion

Comparison of Thyroid Function Changes Across Studies

Multiple observational studies have consistently reported a rise in TSH levels after Ramadan. Or Koca et al. demonstrated a statistically significant increase in median TSH after fasting, without a corresponding decline in free T4, indicating preserved peripheral thyroid hormone availability despite altered absorption dynamics [15]. Similar findings were reported by Ghaffar et al., who reported a significant post-Ramadan increase in TSH levels, especially among patients with short meal-levothyroxine intervals [13]. Similarly, Karoli et al. reported that nearly two-thirds of patients experienced a clinically relevant increase in TSH, which correlated inversely with the interval between levothyroxine intake and meals [14]. In contrast, Zaboon et al. found no significant differences in TSH control among patients taking levothyroxine at pre-Suhoor, pre-Iftar, or post-Iftar times, suggesting that timing alone may not be the dominant determinant of biochemical control [17]. These discrepancies likely reflect variations in adherence, baseline thyroid status, and patient education across cohorts. The results of the pooled meta-analysis support these individual findings by revealing a minor but statistically significant increase in post-Ramadan TSH levels with minimal heterogeneity, indicating consistency in directionality across different populations. Importantly, this chemical shift rarely resulted in overt hypothyroidism.

Timing of Levothyroxine Intake

The results from interventional studies indicated that no single timing strategy was universally superior. Belal et al.'s systematic review revealed that no single timing point (pre-iftar, post-iftar, or pre-suhoor) demonstrated clear superiority in maintaining thyroid control, as all were associated with a significant increase in TSH levels after Ramadan [19], whereas Elsherbiny et al. and Mahzari et al. reported no significant alteration in thyroid function test levels after Ramadan [12,16]. In contrast, another study by El-Kaissi et al. demonstrated that taking levothyroxine 30 minutes before Iftar was associated with the least unfavorable changes in plasma TSH levels after Ramadan [20]. Interestingly, while the post-iftar group reported the highest level of satisfaction, the pre-suhoor group had the highest level of compliance [20].

Conversely, studies documenting short meal-drug intervals have consistently demonstrated inadequate biochemical outcomes. Ghaffar et al. reported significantly higher TSH levels when levothyroxine was taken less than 30 minutes before or after meals, supporting the central role of food-drug interactions rather than clock time per se [13]. These findings are consistent with established levothyroxine pharmacokinetics and support flexible, patient-centered timing strategies that prioritize adequate fasting intervals.

Dose Escalation Versus Alternative Dosing Regimens

Despite these challenges, some studies suggest that specific adjustments, such as increasing the levothyroxine dose by 25-50 g during and 15 days after Ramadan, particularly in high-risk individuals, may help mitigate the observed increase in TSH levels [21]. In a randomized clinical trial, Alzahrani et al. provided high-level evidence on dose adjustments during Ramadan [10]. Preemptive levothyroxine dose escalation (25-50 µg) resulted in considerably lower post-Ramadan TSH levels than did nighttime dosing alone [10]. However, this benefit was outweighed by a significantly higher frequency of subclinical hyperthyroidism, which affected 38.9% of patients [10]. These findings underscore the narrow therapeutic index of levothyroxine and question the safety of routine prophylactic doses. A recent study by Al-Mutawa et al. indicated that an extra dose of levothyroxine (25 g/day) can effectively maintain normal thyroid hormone levels throughout Ramadan, suggesting a potential strategy to prevent the significant post-Ramadan TSH elevation observed in some patients [22]. Furthermore, while an increase in dosage might maintain TSH levels, the implications for T3 and T4 levels and overall patient well-being also require a comprehensive assessment.

Alternative regimens were investigated in smaller randomized and pilot trials. Zakaria and Shahar reported that weekly levothyroxine doses provided biochemical control comparable to that of daily dosing, with no documented side effects and high patient preference [18]. Similarly, Elsherbiny reported that twice- or thrice-weekly dosing maintained euthyroidism at rates comparable to those of daily administration [11]. While promising, these treatments must be validated in larger trials before they can be widely adopted in clinical practice.

Adherence As the Central Determinant of Outcomes

In several studies, adherence to standard levothyroxine administration guidelines emerged as the most dominant and consistent predictor of post-Ramadan thyroid status [12,19,20]. This difficulty is further compounded by the reduced duration of the nocturnal fasting period, making both pre-iftar and pre-suhoor administration problematic for consistent drug absorption [23]. In the IFTAR study, compliant patients had significantly lower post-Ramadan TSH levels and higher rates of euthyroidism compared with nonadherent individuals [12]. This conclusion was consistent across both observational and interventional studies, emphasizing the importance of behavioral considerations over pharmacokinetic complexity in influencing outcomes during Ramadan.

Comparison With Existing Reviews

Systematic evaluations of levothyroxine management during Ramadan remain limited. Prior narrative and systematic syntheses cited in the literature highlighted heterogeneity in study design and concluded that evidence was insufficient to recommend a single optimal dosing strategy. In addition, routine prophylactic dose increase is not recommended due to the risk of subclinical hyperthyroidism, and adjustments should be individualized for the high-risk population.

The present review advances the field by integrating randomized trial data, alternative dosing regimens, and a formal meta-analysis, providing quantitative evidence that fasting during Ramadan results in modest biochemical changes rather than clinically significant instability. Although the observed changes in TSH after Ramadan are likely influenced by multiple confounding factors, such as diet schedule changes, altered sleep schedule, rather than the timing of levothyroxine intake.

Clinical Implications

Collectively, these findings suggest that most hypothyroid patients can safely observe fasting during Ramadan without routine dose escalation, provided that adherence is optimized and food-drug interactions are minimized. Flexible timing strategies tailored to patient preferences are generally safe, whereas dose escalation should be reserved for selected high-risk populations, such as those requiring TSH suppression.

Limitations and Future Directions

The interpretation of these findings is limited by the moderate methodological quality of many included studies, frequent lack of blinding in randomized trials, and short follow-up durations. Another limitation is the heterogeneity in adherence measurement. Future multicenter randomized trials should standardize adherence assessments, include longer post-Ramadan follow-up, and explore alternative levothyroxine formulations with improved absorption profiles. However, further investigations are warranted to evaluate the long-term efficacy and cost-effectiveness of these alternative formulations across diverse fasting populations, including assessments of the accessibility and affordability of liquid and soft-gel formulations for patients in various socioeconomic contexts, particularly in regions where Ramadan fasting is widely practiced [24]. Further research should also explore patient preferences and adherence rates for these novel formulations compared with traditional tablets, as patient compliance remains a critical factor in successful thyroid hormone replacement therapy [23]. Moreover, the included studies have limited geographic concentration, which further limits the applicability of their results. Hence, a holistic understanding of medication adherence during Ramadan extends beyond physiological factors to encompass sociocultural and psychological dimensions that influence patient behavior and treatment outcomes. Therefore, comprehensive strategies must integrate patient education, cultural sensitivity, and personalized treatment plans to optimize outcomes during this unique period [19,25]. Future research should therefore focus on personalized medicine approaches that integrate genetic and metabolic profiling to predict individual responses to altered levothyroxine regimens during Ramadan. This individualized approach could optimize therapeutic outcomes by minimizing the need for reactive dose adjustments and improving patient quality of life.

## Conclusions

In conclusion, Ramadan fasting is associated with a small, consistent increase in TSH among patients with hypothyroidism but rarely results in clinically significant thyroid dysfunction. This observation highlights the physiological impact of fasting on thyroid hormone regulation. Moreover, while the optimal timing for levothyroxine administration during Ramadan is still debated, studies suggest that various schedules can be employed. Patient adherence and convenience remain the cornerstones of successful management, but individualized and flexible levothyroxine regimens can safely accommodate religious observance. Finally, the challenges associated with maintaining euthyroidism in patients with hypothyroidism during Ramadan necessitate a multifactorial approach, incorporating flexible yet effective dosing strategies and considering alternative levothyroxine formulations to mitigate the impact of fasting on absorption kinetics.

## References

[1] Duntas LH, Jonklaas J (2019). Levothyroxine dose adjustment to optimise therapy throughout a patient's lifetime. Adv Ther.

[2] Ernst FR, Barr P, Elmor R (2017). The economic impact of levothyroxine dose adjustments: the CONTROL HE study. Clin Drug Investig.

[3] McMillan M, Rotenberg KS, Vora K (2016). Comorbidities, concomitant medications, and diet as factors affecting levothyroxine therapy: results of the CONTROL surveillance project. Drugs R D.

[4] Caron P, Grunenwald S, Persani L, Borson-Chazot F, Leroy R, Duntas L (2022). Factors influencing the levothyroxine dose in the hormone replacement therapy of primary hypothyroidism in adults. Rev Endocr Metab Disord.

[5] Centanni M, Benvenga S, Sachmechi I (2017). Diagnosis and management of treatment-refractory hypothyroidism: an expert consensus report. J Endocrinol Invest.

[6] Miccoli P, Materazzi G, Rossi L (2020). Levothyroxine therapy in thyrodectomized patients. Front Endocrinol (Lausanne).

[7] Page MJ, McKenzie JE, Bossuyt PM (2021). The PRISMA 2020 statement: an updated guideline for reporting systematic reviews. PLoS Med.

[8] Sterne JA, Savović J, Page MJ (2019). RoB 2: a revised tool for assessing risk of bias in randomised trials. BMJ.

[9] Whiting PF, Rutjes AW, Westwood ME (2011). QUADAS-2: a revised tool for the quality assessment of diagnostic accuracy studies. Ann Intern Med.

[10] Alzahrani AS, Mukhtar N, Alhammad Z (2024). A randomized clinical trial comparing 2 levothyroxine regimens during Ramadan fasting in thyroidectomized patients. J Endocr Soc.

[11] Elsherbiny TM (2022). Twice or thrice weekly versus daily thyroxine in hypothyroid fasting Ramadan: a pilot study. Indian J Endocrinol Metab.

[12] Elsherbiny TM (2023). Impact of fasting on thyrotropin and thyroid status during Ramadan in 292 previously well controlled hypothyroid patients. IFTAR study. Endocrine.

[13] Ghaffar T, Ahmad I, Ahmad Shah Bukhari A (2021). Effect of the timing of thyroxine intake on thyroid stimulating hormone levels in Ramadan. Pak J Med Health Sci.

[14] Karoli R, Fatima J, Chandra A, Mishra PP (2013). Levothyroxine replacement and Ramadan fasting. Indian J Endocrinol Metab.

[15] Or Koca A, Dağdeviren M, Altay M (2020). Should the dose of levothyroxine be changed in hypothyroidism patients fasting during Ramadan?. Turk J Med Sci.

[16] Mahzari M, Al Remthi F, Ajwah I (2023). Levothyroxine timing during Ramadan: a randomized clinical trial. Int J Endocrinol.

[17] Zaboon IA, Alidrisi HA, Hussein IH (2020). Best time for levothyroxine intake in Ramadan (THYRAM): Basrah experience. Int J Endocrinol Metab.

[18] Zakaria NA, Shahar MA (2022). Comparison between weekly vs daily dosing L-thyroxine for the treatment of hypothyroidism in Ramadan-a pilot randomized controlled trial. Malays J Med Health Sci.

[19] Belal MM, Youssef AR, Baker H, Elalaky NA, Marey AA, Quaisy MA, Rabea EM (2024). Effect of Ramadan fasting on thyroid functions in hypothyroid patients taking levothyroxine: a systematic review and meta-analysis. Ir J Med Sci.

[20] El-Kaissi S, AbdelWareth L, Dajani R (2021). Levothyroxine administration during Ramadan: a prospective randomized controlled trial. Eur Thyroid J.

[21] Dellal FD, Ogmen B, Ozdemir D (2020). Effect of Ramadan fasting on thyroid hormone levels in patients on levothyroxine treatment. J Coll Physicians Surg Pak.

[22] Al-Mutawa N, Mussa BM, Akhlaq S, AbdulWahid Z, Qawas A (2025). Extra levothyroxine dose in Ramadan maintained normal thyroid hormone levels in patients with hypothyroidism: a randomized controlled trial. Front Endocrinol (Lausanne).

[23] Al Kaabi J, Afandi B (2024). 12th Al Ain Symposium on Challenges in Diabetes and Endocrinology during Ramadan (virtual meeting), February 16 to 17, 2024. J Diabetes Endocr Pract.

[24] Gunasekaran K, Ng DX, Tan NC (2024). Thyroid function status in patients with hypothyroidism on thyroxine replacement and associated factors: a retrospective cohort study in primary care. BMC Prim Care.

[25] Beshyah SA (2023). Impact of Ramadan fasting on medical conditions: a concise narration of the literature in 2023. Libyan Int Med Univ J.

